# Evaluating NT-proBNP-to-Albumin (NTAR) and RDW-to-eGFR (RGR) Ratios as Biomarkers for Predicting Hospitalization Duration and Mortality in Pulmonary Arterial Hypertension (PAH) and Chronic Thromboembolic Pulmonary Hypertension (CTEPH)

**DOI:** 10.3390/diagnostics15172126

**Published:** 2025-08-22

**Authors:** Dragos Gabriel Iancu, Liviu Cristescu, Razvan Gheorghita Mares, Andreea Varga, Ioan Tilea

**Affiliations:** 1 Doctoral School, George Emil Palade University of Medicine, Pharmacy, Science, and Technology of Targu Mures, 540142 Targu Mures, Romania; dragos-gabriel.iancu@umfst.ro; 2 Faculty of Medicine, George Emil Palade University of Medicine, Pharmacy, Science, and Technology of Targu Mures, 540142 Targu Mures, Romania; razvan.mares@umfst.ro (R.G.M.); ioan.tilea@umfst.ro (I.T.); 3 Faculty of Medicine in English, George Emil Palade University of Medicine, Pharmacy, Science, and Technology of Targu Mures, 540142 Targu Mures, Romania; andreea.varga@umfst.ro

**Keywords:** pulmonary arterial hypertension, chronic thromboembolic pulmonary hypertension, prognostic, biomarkers, length of hospital stay, mortality

## Abstract

**Background/Objectives**: Prognostic biomarkers are essential for guiding the clinical management of pulmonary hypertension (PH). This study aimed to assess both established and novel biomarkers—specifically, the red cell distribution width-to-estimated glomerular filtration rate ratio (RGR) and the NT-proBNP-to-albumin ratio (NTAR)—for their ability to predict length of hospital stay (LOS), prolonged LOS (ELOS), in-hospital mortality, and 3-month all-cause mortality in patients with pulmonary arterial hypertension (PAH) and chronic thromboembolic pulmonary hypertension (CTEPH). **Methods**: A retrospective analysis was conducted on 275 PH-related hospital regular admissions (148 PAH; 127 CTEPH). Established biomarkers—including serum albumin, neutrophil-to-lymphocyte ratio (NLR), Log NT-proBNP, red cell distribution width (RDW), and estimated glomerular filtration rate (eGFR)—as well as novel indices (RGR, and NTAR) were examined for their relationships with LOS, ELOS, in-hospital mortality, and 3-month all-cause mortality. Spearman correlation, univariate logistic regression, and ROC analyses evaluated biomarker relationships and predictive performance. **Results**: Serum albumin independently predicted in-hospital and 3-month mortality in PAH, while in CTEPH, it inversely correlated with LOS and strongly predicted prolonged hospitalization and mortality (AUC = 0.833). NLR had limited correlation with LOS but predicted mortality across both groups. RDW correlated weakly with LOS, significantly predicting prolonged hospitalization (threshold > 52.1 fL) in PAH but not in CTEPH. Preserved renal function (eGFR > 60 mL/min/1.73 m^2^) was inversely associated with LOS in CTEPH patients, suggesting a protective effect. Additionally, reduced eGFR significantly predicted mortality in both PAH (AUC = 0.701; optimal cut-off ≤ 97.4 mL/min/1.73 m^2^) and CTEPH (AUC = 0.793; optimal cut-off ≤ 59.2 mL/min/1.73 m^2^) groups. NTAR (AUC = 0.817) outperformed Log NT-proBNP alone in predicting extended hospitalization and mortality, whereas RGR correlated with LOS and predicted in-hospital mortality. Phenotype-specific analysis demonstrated that inflammatory and renal biomarkers had a stronger prognostic impact in CTEPH. **Conclusions**: Stratification by PH phenotype highlighted the greater prognostic significance of inflammatory and renal indices, particularly in patients with CTEPH. Incorporating NTAR and RGR into clinical workflows may enhance risk stratification and enable more precisely targeted interventions to improve outcomes in pulmonary hypertension.

## 1. Introduction

Pulmonary hypertension (PH) is a complex and multifaceted disease characterized by elevated pulmonary arterial pressure and progressive right ventricular dysfunction, ultimately leading to right heart failure and increased mortality [1].

The estimated global prevalence of PH varies depending on the underlying etiology, affecting approximately 1% of the general population and up to 10% of individuals aged over 65 years [2,3]. Despite therapeutic advancements, PH remains a significant clinical challenge due to its heterogeneous pathophysiology, late diagnosis, and limited therapeutic options [4].

In PH management, length of hospital stay (LOS) serves as a surrogate marker for disease severity, therapeutic efficacy, and healthcare resource utilization. Prolonged LOS (ELOS) is often indicative of complications or suboptimal care strategies [5,6]. Data from the REVEAL Registry indicate a mean LOS of 6.5 days for patients with Group 1 PH (PAH), while analyses of PH admissions via the emergency department report an average LOS of 6.9 days [7,8].

Hypoalbuminemia is a frequent manifestation in PH, reflecting systemic inflammation and endothelial dysfunction that increase capillary permeability and promote fluid extravasation into the lungs, myocardium, and splanchnic vasculature. Reduced serum albumin also alters the pharmacokinetics of loop diuretics, contributing to diuretic resistance and worsening hemodynamic status. Beyond its pathophysiological roles, hypoalbuminemia often signals underlying malnutrition or chronic inflammatory states—both established predictors of prolonged hospitalization and higher mortality. In a retrospective cohort of 163 PAH patients, low albumin levels independently predicted longer hospital stays, higher morbidity, and increased risk of death [9,10,11,12,13].

The neutrophil-to-lymphocyte ratio (NLR)—a simple, cost-effective marker of systemic inflammation—has demonstrated robust prognostic value in pulmonary hypertension [14]. Elevated NLR is consistently linked to longer hospital stays, higher rates of morbidity, and increased all-cause mortality [15,16]. In a large multicenter study among PH cohort patients, an NLR above the cohort median independently predicted both early and long-term mortality [17]. These findings support the incorporation of NLR into routine risk stratification algorithms, facilitating early identification of high-risk patients.

The 2022 ESC/ERS Guidelines for the Diagnosis and Treatment of Pulmonary Hypertension highlight NT-proBNP as a pivotal biomarker in evaluating right ventricular function and overall prognosis in PH patients [1]. Due to its longer plasma half-life, NT-proBNP is a stable indicator of cardiac dysfunction [18]. It provides valuable insights into disease severity and progression and is influenced by age and sex, showing higher concentrations in older individuals and females [19,20].

Red cell distribution width (RDW)—an index of erythrocyte size variability (anisocytosis)—has been linked to adverse outcomes across cardiovascular and pulmonary diseases. In PH, elevated RDW is associated with increased mortality and prolonged hospital stays. Similar associations have been noted in chronic obstructive pulmonary disease (COPD) patients with concomitant PH [21,22,23]. Importantly, Hampole et al. demonstrated that RDW independently predicts mortality in PH patients and outperforms NT-proBNP in prognostic accuracy [24].

Renal function, as measured by the estimated glomerular filtration rate (eGFR), is a critical determinant of clinical outcomes in patients with PAH [25]. Data from the REVEAL Registry demonstrated that a decline in eGFR of 10% or more over a year independently predicted poorer survival in PAH patients [26]. Additionally, a study conducted by Shah et al. found that serum creatinine and eGFR were independent risk predictors of mortality in PAH patients [27].

The aim of this study was to evaluate the prognostic performance of two novel laboratory-based biomarker ratios—the red cell distribution width-to-eGFR ratio (RGR) and the NT-proBNP-to-albumin ratio (NTAR)—for predicting LOS, ELOS, in-hospital mortality, and 3-month all-cause mortality in patients with pulmonary arterial hypertension and chronic thromboembolic pulmonary hypertension. Their discriminative accuracy was benchmarked against five established biomarkers (albumin, NLR, NT-proBNP, RDW, and eGFR). Multivariable logistic regression analyses were then conducted to identify independent prognostic indicators.

## 2. Materials and Methods

### 2.1. Study Design and Population

This retrospective observational study was carried out in the Department of Internal Medicine II—Cardiology at the County Emergency Clinical Hospital Targu Mures, Romania, from 1 September 2015 to 31 October 2024. To the best of our knowledge, it is the first European investigation to evaluate the prognostic utility of two novel biomarker ratios in pulmonary hypertension—NTAR and RGR—in relation to hospital LOS, ELOS, in-hospital mortality, and 3-month all-cause mortality. These exploratory indices were benchmarked against established prognostic markers to determine their incremental predictive value. The analytic cohort comprised 468 consecutive PH-related admissions, identified through the hospital’s electronic information system. As a complete census of our registry, no sample size calculation was performed [28].

### 2.2. Inclusion and Exclusion Criteria

All consecutive hospital admissions of patients who met the ESC/ERS 2015 or 2022 criteria for PAH or CTEPH and remained hospitalized for at least 48 h were eligible for inclusion [1,29].

Admissions complicated by active inflammation or infection (e.g., sepsis), solid or hematologic malignancies (including myelodysplastic syndromes), autoimmune disorders, primary or infectious liver disease, or end-stage renal disease (CKD stage V) were ineligible, as were records with incomplete clinical data. After applying these criteria, the study cohort consisted of 275 admissions, of which 148 were PAH and 127 were CTEPH.

### 2.3. Treatment, Management, and Disease Severity Parameters

All patients received guideline-directed medical therapy in line with the current ESC/ERS recommendations for PAH and CTEPH, ensuring consistent treatment exposure across the cohort. Disease severity was quantified by WHO-FC and circulating NT-proBNP concentrations to capture both functional impairment and hemodynamic stress. Extended length of stay (ELOS) was defined as a hospitalization exceeding seven days.

### 2.4. Laboratory Assessments

Venous blood was collected after an 8-hour fast and within two hours of hospital admission. Complete blood counts—including total white blood cell (WBC) count with neutrophil and lymphocyte differentials—were performed on a Sysmex XN-550 analyzer (Sysmex Corporation, Kobe, Japan). Serum biochemistry was measured using the Konelab Prime 60i system (Thermo Fisher Scientific Inc., Waltham, MA, USA), and NT-proBNP concentrations were quantified with the Nano-Checker™ 710 Reader (Nano-Ditech Corporation, Cranbury, NJ, USA). All assays were carried out in an ISO 15189-accredited laboratory.

### 2.5. Comorbidities

Comorbidity conditions were systematically recorded and their prevalence calculated separately for the PAH and CTEPH cohorts.

Documented cardiovascular disorders were systemic hypertension, coronary artery disease, atrial fibrillation, and prior deep vein thrombosis (DVT). Metabolic conditions such as type II diabetes mellitus (T2DM) and thyroid dysfunction were noted, while respiratory diagnoses included obstructive sleep apnea, asthma, COPD, and other chronic lung diseases. A documented history of SARS-CoV-2 infection was also considered.

### 2.6. LOS, ELOS, and Mortality Assessment

Based on the REVEAL Registry, which reports a mean LOS of 6.9 days, we defined ELOS in our study as a hospitalization duration exceeding 7 days [7,8].

Mortality outcomes were evaluated for both in-hospital mortality and post-discharge (3 months). In-hospital mortality was defined as any death occurring during the index hospital admission. Post-discharge mortality was assessed through electronic medical records and structured phone calls with patients’ families or caregivers, in compliance with the General Data Protection Regulation (GDPR).

### 2.7. Proposed New Biomarkers

In addition to established laboratory parameters, two novel, unit-independent indices were introduced to enhance prognostic stratification—the RDW-to-eGFR ratio (RGR) and the NT-proBNP-to-albumin ratio (NTAR). Both indices are calculated as simple quotients of routinely measured biomarkers, yielding dimensionless metrics that obviate unit conversion challenges and support seamless incorporation into clinical risk models. Detailed calculation methods and analytical performance for RGR and NTAR are outlined in the subsequent sections.

#### 2.7.1. RDW-SD-to-eGFR Ratio (RGR)

Renal impairment reduces erythropoietin production, leading to increased anisocytosis and elevated RDW. The estimated glomerular filtration rate—calculated using the 2021 CKD-EPI equation—served as the measure of renal function.

The RGR reflects both anisocytosis and ineffective erythropoiesis—often driven by chronic inflammation, hypoxemia, or nutritional deficiencies—and renal function, as indicated by the eGFR. Renal impairment is common in PH and is strongly associated with adverse outcomes, primarily due to reduced renal perfusion and venous congestion resulting from right heart failure. Elevated RDW has been independently linked to poor prognosis in PH, while reduced eGFR signifies systemic end-organ dysfunction. Therefore, RGR captures the interplay between hematologic abnormalities and renal dysfunction—two key systems involved in the progression of PH. RGR was derived by dividing the RDW standard deviation (measured in femtoliters, fl) by eGFR (mL/min/1.73 m^2^):

$$RGR=\frac{RDW\;standard\;deviation\;(\mathrm{fl})}{eGFR\;(\mathrm{mL}/\min/1.73\;m^{2})}$$

#### 2.7.2. NT-proBNP-to-Albumin Ratio (NTAR)

The NT-proBNP-to-albumin ratio (NTAR) is an emerging biomarker that reflects both myocardial wall stress and systemic congestion in PH. Its underlying mechanism is rooted in the complex interaction between cardiac overload and hepatic dysfunction. Elevated right ventricular strain stimulates the release of natriuretic peptides, particularly NT-proBNP, while progressive PH leads to hepatic congestion, disrupting cellular homeostasis and impairing albumin synthesis. NTAR was determined as the base-10 logarithm of the NT-proBNP-to-albumin ratio:

$$NTAR=log(10)\frac{NT-proBNP\;(\frac{pg}{mL})}{Albumin\;(\frac{g}{dL})}$$

### 2.8. Statistical Analysis

Descriptive and analytical statistics for the final dataset was performed using MedCalc^®^ Statistical Software (version 23.1.6, MedCalc Software Ltd., Ostend, Belgium; https://www.medcalc.org; accessed on 30 March 2025). A *p*-value of <0.05 was considered the threshold for statistical significance.

Continuous variables were tested for normality using the Kolmogorov–Smirnov test. Parametric continuous variables are presented as the mean ± standard deviation (SD), while non-parametric continuous variables are reported as the median and interquartile range (IQR). Categorical variables are expressed as counts (percentages).

Between-group comparisons of continuous variables were performed using Student’s t-test when data followed a normal distribution and the Mann–Whitney U test for non-normally distributed data. Relationships between continuous measures were quantified using Spearman’s rank correlation coefficient for non-parametric variables.

Logistic regression was applied to classify data into binary variables (defined as 1 for ELOS, in-hospital, and 3-month all-cause mortality). The odds ratio (OR) along with the 95% confidence interval (CI) was computed to evaluate the relationship between predictors and outcomes. Model goodness-of-fit was determined using the Hosmer–Lemeshow test, indicated by a *p*-value greater than 0.05.

To assess predictive accuracy, a receiver operating characteristic (ROC) curve analysis was conducted, and the area under the curve (AUC) was determined with a 95% CI. The AUC was interpreted according to the classification proposed by Çorbacıoğlu and Aksel [30]. Comparisons between ROC curves were carried out using DeLong’s test. Optimal cut-off values were identified based on the Youden index.

Analyzed data are presented raw and unadjusted for confounders.

## 3. Results

### 3.1. Characteristics of the Study Cohort

Of the 468 pulmonary hypertension-related hospital admissions initially identified, 275 met the predefined inclusion and exclusion criteria and were included in the analysis. Among these, 148 (53.8%) admissions were identified as PAH and 127 (46.2%) as CTEPH. The PAH cohort demonstrated a higher proportion of female patients, whereas sex distribution in the CTEPH group was more balanced. Functional limitation was primarily moderate to severe, with most participants being assigned WHO-FC II or III. Within the PAH subgroup, congenital heart disease-associated PAH (CHD-PAH) was the most common etiology, accounting for 102 patients (68.92%), followed by idiopathic PAH (IPAH) with 21 patients (14.19%), connective tissue disease-associated PAH (CTD-PAH) with 20 patients (13.51%), and portopulmonary hypertension (PoPH) with 5 patients (3.38%). Key demographic and clinical characteristics are presented in Table 1.

### 3.2. Comorbidities

Comorbidity profiles differed substantially between the PAH and CTEPH groups. Excess body weight was common in both cohorts. Systemic hypertension and COPD were more prevalent in the CTEPH group. A history of DVT was observed almost exclusively in CTEPH patients (91.34%), consistent with its known pathophysiological basis. In contrast, atrial fibrillation and thyroid disease were more frequently encountered in the PAH group. The prevalence of T2DM and a prior SARS-CoV-2 infection was comparable between the two groups.

### 3.3. Length of Hospital Stay and Extended Length of Hospital Stay

The median length of hospital stay (LOS) was 7 days in both groups. Extended length of stay (ELOS) was observed in 41.22% of PAH patients and 45.67% of those with CTEPH, reflecting a substantial burden of hospitalization. Notably, patients requiring ELOS were significantly more likely to present with advanced functional limitation (WHO-FC III–IV) compared with those with shorter admissions (≤7 days; *p* = 0.001 in both groups), suggesting an association between clinical severity and prolonged hospitalization.

### 3.4. Laboratory Data

A total of seven biomarkers were evaluated across the study population, encompassing parameters derived from hematological indices and routine biochemical measurements. These markers were selected based on their potential relevance in pulmonary hypertension pathophysiology. An overview of the biomarker values distribution in PAH and CTEPH groups is provided in Table 2.

### 3.5. Biomarkers and LOS

Analysis by PH subtype (Spearman correlations) revealed distinct biomarker associations with LOS. In the CTEPH cohort, serum albumin and eGFR showed negative correlations with LOS, indicating that hypoalbuminemia and reduced renal function were linked to prolonged hospitalization in this subgroup. These associations were not statistically significant in the PAH group. In contrast, positive correlations with LOS were consistently observed across both groups for Log NT-proBNP, NTAR, NLR, RGR, and RDW, reflecting their potential role as markers of disease severity and hospitalization burden. These correlations were consistently stronger in the CTEPH group (Table 3).

### 3.6. Biomarkers and ELOS

To identify predictors of ELOS within each phenotype group, univariate logistic regression analysis was performed (Table 4). In the PAH group, NLR, RDW, RGR, NTAR, and Log NT-proBNP showed weak but statistically significant associations with ELOS, based on AUC values and satisfactory model calibration. Among these, NLR and RDW exhibited the highest AUCs (0.647 and 0.642, respectively). In the CTEPH group, Log NT-proBNP, NTAR, RGR, NLR, and eGFR emerged as significant predictors of ELOS, with Log NT-proBNP and NTAR again demonstrating the strongest discriminative performance (AUCs: 0.748 and 0.743, respectively).

These findings support the potential clinical utility of cardiac stress markers (NT-proBNP, NTAR), inflammatory indices (NLR, RGR), and red cell distribution measures (RDW) in identifying patients at increased risk of prolonged hospitalization. In patients with CTEPH, reduced eGFR also emerged as an independent predictor of ELOS.

**Figure 1:**
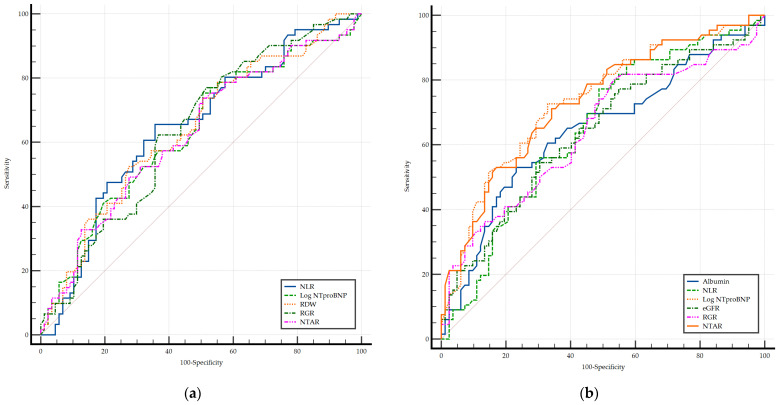
Comparison of the ROC curves for ELOS as the dependent value expected in PAH (**a**) and CTEPH (**b**) groups. eGFR, estimated glomerular filtration rate; Log NT-proBNP, logarithm of the NT-proBNP; NLR, neutrophil-to-lymphocyte ratio; NTAR, NT-proBNP-to-albumin ratio; RDW, red cell distribution width; RGR, red cell distribution width-to-eGFR ratio.

An ROC curve analysis was conducted to compare the relative performance of the biomarkers in predicting ELOS (Figure 1). In PAH, no significant differences were observed between the markers in terms of discriminatory power. In contrast, in CTEPH, Log NT-proBNP and NTAR demonstrated significantly greater AUCs compared with albumin, NLR, eGFR, and RGR (*p* < 0.001), although no significant difference was found between Log NT-proBNP and NTAR themselves.

### 3.7. Biomarkers and In-Hospital Mortality

Twelve deaths occurred (eight PAH, four CTEPH), and the male-to-female death ratio was 1:1.2. All were WHO-FC III-IV patients at the time of admission. Univariate logistic regression identified multiple significant predictors of in-hospital mortality (Table 5).

In the PAH group, several biomarkers demonstrated strong predictive value for in-hospital mortality. NLR, NTAR, and albumin exhibited good predictive capacity, while Log NT-proBNP, RGR, eGFR, and RDW showed acceptable discrimination. These findings were supported by a well-calibrated model, as indicated by the Hosmer–Lemeshow test. Notably, higher serum albumin levels emerged as a significant protective factor.

In CTEPH, NTAR demonstrated the highest predictive performance for in-hospital mortality (AUC = 0.817), indicating good discriminative ability. A very similar AUC, considered acceptable, was observed for Log NT-proBNP, followed by albumin and NLR. Poor discriminatory power was noted for eGFR and RGR, with RGR showing the lowest AUC.

**Figure 2:**
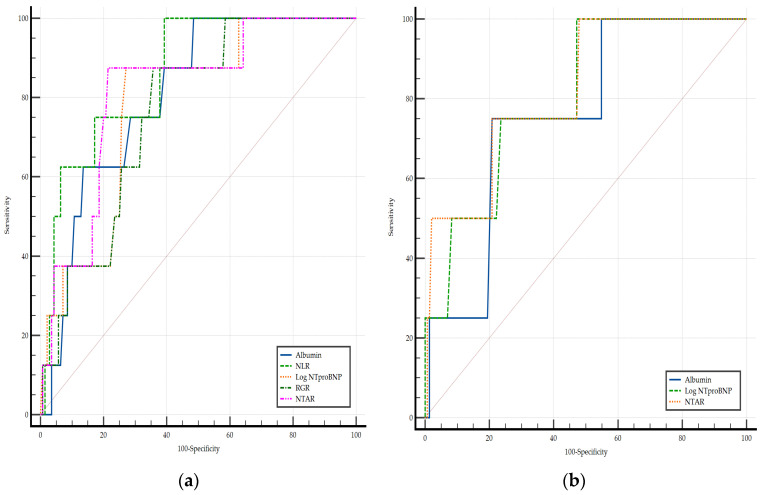
Comparison of the ROC curves for in-hospital mortality as the dependent value expected in PAH (**a**) and CTEPH (**b**) groups. Log NT-proBNP; NLR, neutrophil-to-lymphocyte ratio; NTAR, NT-proBNP-to-albumin ratio; RGR, red cell distribution width-to-eGFR ratio.

In the ROC curve analysis comparison, both in Figure 2a,b, there were no significant differences in terms of AUC in the leading biomarkers.

### 3.8. Biomarkers and 3-Month All-Cause Mortality

In the PAH cohort, univariate logistic regression analysis identified serum albumin as the only statistically significant predictor of 3-month all-cause mortality (AUC = 0.782), with lower concentrations being independently associated with increased mortality risk. In the CTEPH group, both NLR and serum albumin demonstrated very good prognostic performance for predicting 3-month all-cause mortality, with AUCs of 0.875 and 0.833, respectively.

These findings suggest a phenotype-specific prognostic pattern for 3-month all-cause mortality, where hypoalbuminemia is most relevant in the PAH group, while elevated NLR and low albumin emerge as the dominant predictors in the CTEPH group (Table 6). Additionally, reduced eGFR demonstrated good predictive capacity in this subgroup.

**Figure 3:**
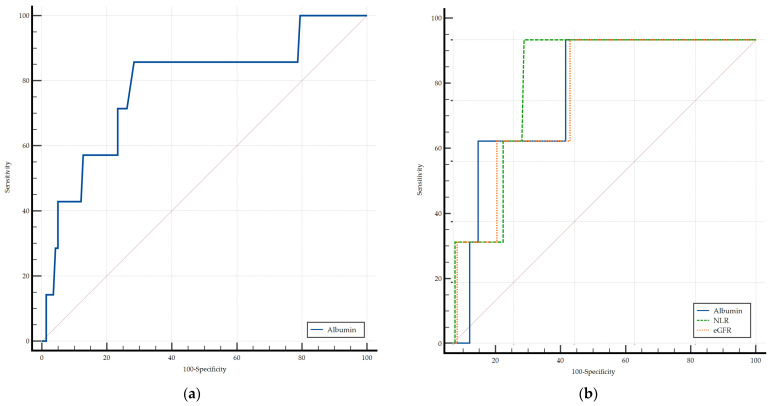
Comparison of ROC curves for 3-month all-cause mortality as the dependent value expected in PAH (**a**) and CTEPH (**b**) groups. eGFR, estimated glomerular filtration rate; NLR, neutrophil-to-lymphocyte ratio.

For the prediction of 3-month all-cause mortality, serum albumin was the only biomarker in the PAH group that demonstrated statistically significant discriminative ability and was thus selected for ROC analysis (Figure 3a). In the CTEPH group, albumin, NLR, and eGFR were included in the comparison; however, no statistically significant differences were observed between their respective AUCs (Figure 3b).

## 4. Discussion

In this study, we evaluated the prognostic significance of both established and novel biomarkers in relation to hospitalization outcomes and mortality across 275 PH-related hospital admissions, stratified by clinical phenotype (PAH and CTEPH). Our findings underscore the utility of a composite biomarker strategy—encompassing inflammatory, hematologic, cardiac, and renal indicators—tailored to the underlying disease profile. Notably, this is the first investigation to propose and validate RGR and the NTAR as prognostic markers in this context. NTAR demonstrated particularly strong discriminative performance in CTEPH patients, especially for ELOS and in-hospital mortality, whereas RGR showed weaker standalone performance but may offer added prognostic value in future multimodal assessment models.

### 4.1. Prognostic Utility of Conventional Biomarkers and Cut-Off Determination

In our analysis, serum albumin exhibited distinct phenotype-specific prognostic patterns in the PAH and CTEPH groups. In PAH patients, lower albumin concentrations were significantly associated with increased in-hospital and 3-month all-cause mortality, respectively, but showed no meaningful association with LOS or ELOS. Conversely, in CTEPH patients, albumin demonstrated a moderate yet significant negative correlation with LOS and emerged as a predictor of ELOS. It also showed good and very good discriminative performance for in-hospital and 3-month mortality, with AUCs of 0.772 and 0.833, respectively. These results underscore the prognostic relevance of hypoalbuminemia as phenotype-dependent: primarily mortality-driven in PAH, and more closely associated with hospitalization burden in CTEPH. Our findings are consistent with prior evidence linking hypoalbuminemia to both adverse clinical outcomes and prolonged hospitalizations [31,32,33,34].

NLR, a widely studied marker of systemic inflammation, demonstrated a weak yet statistically significant correlation with LOS exclusively in the CTEPH group. It did not reliably predict ELOS in PAH patients, although it reached statistical significance in the CTEPH group, albeit with limited discriminative capacity. Its prognostic utility was more robust in relation to mortality outcomes. In PAH patients, an NLR value > 3.28 was strongly associated with increased in-hospital mortality risk. In the CTEPH group, NLR thresholds >2.96 and >4.83 were predictive of in-hospital and 3-month all-cause mortality, respectively. These findings are in line with previous studies: Özpelit et al. identified an NLR cut-off of 3.0 for predicting all-cause mortality in PAH patients, while Yanartaş et al. reported that an NLR > 2.54 was significantly associated with increased postoperative mortality in CTEPH [35,36].

Log NT-proBNP demonstrated a moderate positive correlation with LOS in both PAH and CTEPH groups, with a stronger association observed in the CTEPH group. It emerged as a significant predictor of ELOS in both phenotypes, exhibiting the highest discriminative performance in CTEPH. Regarding mortality, elevated Log NT-proBNP values were associated with increased in-hospital mortality in PAH and showed similarly good predictive performance in CTEPH. For 3-month all-cause mortality, its prognostic utility was limited in PAH but good in CTEPH. These findings are supported by previous studies: Maurer et al. identified a threshold of 2.73 as predictive of all-cause mortality in PH patients, while Januzzi et al. reported that Log NT-proBNP levels exceeding 2.99 were associated with a 2.88-fold increase in one-year mortality among individuals with heart failure [37,38]. Clinically, each one-unit increase in Log NT-proBNP—reflecting a tenfold rise in NT-proBNP concentration—was associated with prolonged hospitalization and greater short-term mortality risk, particularly in patients with CTEPH.

RDW exhibited a weak positive correlation with LOS in both PAH and CTEPH patients, suggesting its potential utility as a marker of disease severity. In the PAH group, RDW was significantly associated with an increased likelihood of ELOS. Conversely, RDW demonstrated limited prognostic value in the CTEPH group, showing no significant association with either ELOS or mortality. These findings align closely with prior research, including a large-scale study of 167,292 admissions, which reported that elevated RDW at hospital admission was associated with longer stays compared with admissions with lower RDW values [39]. Elevated RDW has also been widely recognized as a predictor of in-hospital mortality and all-cause death across multiple patient populations [40,41]. Nevertheless, in our cohort, the association between RDW and in-hospital mortality was marginal, suggesting the need for further validation in larger, prospective studies.

eGFR demonstrated a significant negative correlation with LOS in the CTEPH group and independently predicted ELOS, with values > 60 mL/min/1.73 m^2^ conferring a protective effect. In the PAH group, lower eGFR was associated with higher in-hospital mortality risk, while in the CTEPH group, it exhibited good discriminative performance for 3-month all-cause mortality. These results underscore the prognostic importance of renal dysfunction across PH phenotypes, particularly in CTEPH. Prior investigations have similarly linked reduced eGFR to prolonged hospitalizations and increased ICU admissions; reported a 15% rise in 30-day mortality per 10 mL/min/1.73 m^2^ decline in heart failure cohorts; and identified eGFR < 56 mL/min/1.73 m^2^ as an independent predictor of in-hospital death in patients with pneumonia [42,43,44]. Together, these data highlight the critical role of preserving renal function to mitigate mortality and reduce hospitalization duration.

### 4.2. RGR and NTAR in Focus: New Predictive Tools for Risk Stratification

RGR, a novel composite biomarker, demonstrated consistent prognostic utility across PAH and CTEPH phenotypes. RGR exhibited a positive correlation with LOS in both cohorts, with a stronger effect size in CTEPH. As a predictor of ELOS, RGR achieved acceptable discrimination in PAH and CTEPH. For in-hospital mortality, RGR showed good discriminative performance in PAH and maintained acceptable accuracy in CTEPH. Elevated RGR likely reflects combined disturbances in erythropoiesis and renal function—manifested as increased anisocytosis alongside reduced glomerular filtration—underscoring the interplay between hematologic variability and renal impairment in PH prognostication [45]. These results corroborate earlier studies linking RDW variability to adverse PH outcomes and highlight the integral role of cardiorenal dysfunction in shaping patient prognosis [46,47,48].

The NTAR demonstrated robust prognostic value in both the PAH and CTEPH cohorts. NTAR correlated moderately with LOS—more strongly in the CTEPH group—and was significantly predictive of ELOS in both phenotypes. Its discriminative performance for in-hospital mortality was particularly high, with NTAR emerging as an independent risk factor in PAH patients and showing borderline significance in the CTEPH group. These data indicate that NTAR serves as a dual indicator of hospitalization burden and short-term mortality in PAH and CTEPH. Prior studies have linked hypoalbuminemia—an important marker of nutritional status and a marker of illness—to poorer outcomes in hospitalized patients and identified low serum albumin as a marker of higher mortality risk in PAH [13,49,50].

While NTAR and RGR have demonstrated strong prognostic associations in PH, a more in-depth discussion of the pathophysiological mechanisms through which these biomarkers influence disease progression would be beneficial. Elevated NTAR likely reflects a convergence of increased myocardial wall stress—driving NT-proBNP release—and impaired albumin synthesis due to hepatic congestion or chronic systemic inflammation, thereby compounding right ventricular dysfunction. Conversely, an elevated RGR embodies the dual impact of erythropoietic disruption (manifested as anisocytosis) and renal impairment, conditions that potentiate hypoxia-driven pulmonary vascular remodeling and endothelial dysfunction, ultimately escalating pulmonary vascular resistance [51]. A deeper exploration of these mechanisms could refine risk stratification models and identify novel targets for therapeutic intervention in PH.

These analyses highlight the complex pathophysiological interdependence among cardiorenal dysfunction, systemic inflammation, and metabolic dysregulation in the prognostication of prolonged hospitalizations and mortality, underscoring the imperative for a precision medicine approach integrating multimodal biomarker profiling to enhance risk stratification and therapeutic optimization across the continuum of pulmonary arterial hypertension and chronic thromboembolic pulmonary hypertension progression.

### 4.3. Limitations and Future Directions

While this study provides valuable preliminary insights, its retrospective, single-center design introduces inherent limitations. The reliance on a single institution’s electronic records may engender selection bias and constrain the generalizability of our findings to broaden PH populations. Moreover, the absence of invasive hemodynamic measurements—such as right heart catheterization-derived pulmonary artery pressures and pulmonary vascular resistance—precludes direct mechanistic correlations with NTAR and RGR. Biomarker sampling was limited to admission values, preventing the assessment of temporal dynamics, and the cohort size (275 admissions) lacked sufficient power for granular subgroup analyses by etiology, comorbidity burden, or specific therapeutic regimens.

Future research should emphasize large-scale, prospective multicenter studies to validate and expand upon these findings. Inclusion of geographically diverse pulmonary hypertension centers would help reduce selection bias and enhance external validity. Longitudinal biomarker assessments—ideally at baseline and at 1, 3, and 6 months of follow-up—could clarify how NTAR and RGR trajectories correlate with clinical outcomes over time. Incorporating invasive hemodynamic measurements and advanced imaging techniques (e.g., speckle-tracking echocardiography, three-dimensional echocardiography, cardiac magnetic resonance imaging, computed tomography, and hybrid imaging) would provide valuable insights into the pathophysiological mechanisms linking these biomarker ratios to right ventricular function and pulmonary vascular remodeling.

Our findings also underscore the need to translate NTAR and RGR from research metrics into practical clinical tools. Embedding automated alerts for high-risk thresholds (e.g., NTAR > 3.0; RGR > 0.6) within electronic health records could prompt timely multidisciplinary interventions, such as intensified decongestive therapy, nutritional support, or early nephrology referral. Additionally, targeted analyses of how specific PH treatments (prostacyclin analogs, endothelin receptor antagonists, PDE-5 inhibitors) modulate these biomarkers will refine their interpretive context. Finally, health economic evaluations comparing standard management to NTAR/RGR-guided pathways are essential to determine cost-effectiveness, resource utilization, and potential improvements in patient-centered outcomes.

## 5. Conclusions

The NT-proBNP-to-albumin ratio (NTAR) and the red cell distribution width-to-eGFR ratio (RGR) consistently predicted prolonged hospitalization and in-hospital mortality, with especially strong performance in the CTEPH phenotype. By capturing the interplay between cardiac stress, systemic inflammation, and renal function in a single metric, these novel indices augment traditional risk models and facilitate more nuanced patient stratification. To transition from proof-of-concept to clinical practice, large-scale, prospective multicenter validation is essential. Such efforts should assess NTAR and RGR across diverse PH groups, explore integration with hemodynamic and imaging data, and evaluate their impact on management algorithms. Ultimately, embedding these biomarkers into routine care pathways holds the promise of earlier risk identification and individualized therapeutic interventions, with the goal of improving outcomes in pulmonary arterial hypertension and chronic thromboembolic pulmonary hypertension.

## Figures and Tables

**Table 1:** Baseline characteristics of the PAH and CTEPH admissions.

Parameter	PAH (*n* = 148)	CTEPH (*n* = 127)
Age (years, median, IQR)	51 (42.00–67.00)	67 (57.50–71.00)
Sex (female, *n*, %)	84 (56.75)	57 (44.88)
BMI (kg/m^2^, median, IQR)	28.16 (23.20–32.3)	26.7 (24.64–31.14)
WHO-FC (*n*, %)		
I	8 (5.41)	8 (6.30)
II	50 (33.78)	53 (41.73)
III	72 (48.65)	46 (36.22)
IV	18 (12.16)	20 (15.75)
Comorbidities (*n*, %)		
Cardiovascular Diseases		
Systemic hypertension	47 (31.76)	51 (40.16)
Coronary artery disease	11 (22.45)	19 (14.96)
Atrial fibrillation	49 (33.11)	24 (18.90)
Previous deep vein thrombosis	-	116 (91.34)
Metabolic Disorders		
Excess body weight	97 (65.54)	89 (70.08)
T2DM	50 (33.78)	38 (29.92)
Thyroid disease	40 (27.03)	23 (18.11)
Respiratory Diseases		
Obstructive sleep apnea	14 (9.46)	13 (10.24)
Asthma	11 (7.43)	7 (5.51)
COPD	10 (6.76)	20 (15.75)
Lung disease—other than COPD and asthma	52 (35.14)	28 (22.05)
History of SARS-CoV-2 infection	62 (41.90)	54 (42.52)
Length of hospital stay		
LOS (days, median, IQR)	7 (5.00–9.00)	7 (5.00–9.00)
ELOS (*n*, %)	61 (41.22)	58 (45.67)
ELOS (days, median, IQR)	10 (8.00–15.00)	14 (8.00–15.75)
Mortality (*n*, %)		
Overall mortality	15 (10.14)	7 (5.51)
In-hospital mortality	8 (5.41)	4 (3.15)
3-month all-cause mortality	7 (4.73)	3 (2.36)

BMI, body mass index; COPD, chronic obstructive pulmonary disease; CTEPH, chronic thromboembolic pulmonary hypertension; ELOS, extended length of hospital stay; IQR, interquartile range; LOS, length of hospital stay; *n*, number of admissions; PAH, pulmonary arterial hypertension; T2DM, type 2 diabetes mellitus; WHO-FC, World Health Organization functional classification.

**Table 2:** Distribution of established and novel biomarker indices in PAH and CTEPH groups.

Parameter	PAH (*n* = 148)	CTEPH (*n* = 127)
Laboratory data		
Neutrophils (×10^3^/µL, mean ± SD)	4.92 ± 2.44	4.88 ± 1.62
Lymphocytes (×10^3^/µL, median, IQR)	1.56 (1.13–2.13)	1.39 (0.97–1.77)
Creatinine (mg/dL, mean ± SD)	0.98 ± 0.38	1.23 ± 0.52
NT-proBNP (pg/mL, mean ± SD)	3120.26 ± 3702.10	4534.54 ± 4926.93
Prior studied biomarkers		
Albumin (g/dL, median, IQR)	4.14 (3.82–4.48)	4.15 (3.83–4.38)
NLR (mean ± SD)	3.57 ± 2.25	4.07 ± 2.47
Log NT-proBNP (median, IQR)	3.22 (2.60–3.68)	3.4 (2.81–3.84)
RDW (fl, mean ± SD)	51.31 ± 9.75	51.88 ± 8.20
eGFR (ml/min/1.73 m^2^, median, IQR)	85.1 (63.62–110.32)	63.19 (47.29–90.01)
Proposed biomarkers		
RGR (mean ± SD)	0.85 ± 2.36	0.91 ± 0.50
NTAR (median, IQR)	2.62 (1.95–3.08)	2.79 (2.21–3.23)

CTEPH, chronic thromboembolic pulmonary hypertension; eGFR, estimated glomerular filtration rate; IQR, interquartile range; *n*, number; NTAR, NT-proBNP-to-albumin ratio; PAH, pulmonary arterial hypertension; RDW, red cell distribution width; RGR, red cell distribution width-to-eGFR ratio.

**Table 3:** Phenotype-specific Spearman correlations of biomarkers with length of hospitalization.

Parameters	Length of Hospital Stay (LOS)			
	PAH		CTEPH	
	*r*	*p*	*r*	*p*
Prior studied biomarkers				
Albumin	−0.13	0.122	−0.24	0.007
NLR	0.23	0.004	0.33	<0.001
Log NT-proBNP	0.23	0.005	0.48	<0.001
RDW	0.21	0.009	0.19	0.028
eGFR	−0.10	0.216	−0.31	<0.001
Proposed biomarkers				
RGR	0.20	0.016	0.33	<0.001
NTAR	0.23	0.005	0.46	<0.001

CTEPH, chronic thromboembolic pulmonary hypertension; eGFR, estimated glomerular filtration rate; NLR, neutrophil-to-lymphocyte ratio; NTAR, NT-proBNP-to-albumin ratio; PAH, pulmonary arterial hypertension; RDW, red cell distribution width; RGR, red cell distribution width-to-eGFR ratio.

**Table 4:** Univariate logistic regression analysis of predictors for ELOS in PAH and CTEPH groups.

Independent Variables	Extended Length of Hospital Stay (ELOS)					
	AUC (95% CI)	*p*	OR (95% CI)	*p*	Hosmer–Lemeshow Test, *p*	Associated Criterion to Youden Index J
**PAH group**						
Prior studied biomarkers						
Albumin	0.579 (0.495–0.659)	0.102	0.587 (0.301–1.146)	0.118	0.449	≤4.18
NLR	0.647 (0.564–0.723)	0.001	1.150 (0.989–1.338)	0.068	0.095	>3.11
Log NT–proBNP	0.630 (0.547–0.708)	0.006	1.753 (1.098–2.800)	0.018	0.506	>3.00
RDW	0.642 (0.559–0.719)	0.002	1.049 (1.011–1.089)	0.010	0.659	>52.10
eGFR	0.580 (0.496–0.660)	0.096	0.990 (0.980–1.000)	0.061	0.896	≤84.11
Proposed biomarkers						
RGR	0.637 (0.554–0.715)	0.002	3.722 (1.187–11.671)	0.024	0.084	>0.60
NTAR	0.627 (0.544–0.705)	0.007	1.773 (1.116–2.817)	0.015	0.800	>2.37
**CTEPH group**						
Prior studied biomarkers						
Albumin	0.640 (0.550–0.723)	0.006	1.031 (0.890–1.195)	0.680	0.012	≤3.95
NLR	0.645 (0.555–0.727)	0.003	1.208 (1.035–1.410)	0.016	0.667	>2.77
Log NT-proBNP	0.748 (0.664–0.821)	<0.001	3.947 (2.085–7.471)	<0.001	0.510	>3.47
RDW	0.551 (0.460–0.639)	0.333	1.030 (0.986–1.075)	0.180	0.139	>52.20
eGFR	0.645 (0.555–0.728)	0.003	0.984 (0.972–0.997)	0.014	0.766	≤60.00
Proposed biomarkers						
RGR	0.648 (0.559–0.731)	0.003	3.766 (1.582–8.960)	0.002	0.309	>0.58
NTAR	0.743 (0.658–0.817)	<0.001	3.641 (1.992–6.654)	<0.001	0.979	>3.09

AUC, area under the curve; CI, confidence interval; CTEPH, chronic thromboembolic pulmonary hypertension; eGFR, estimated glomerular filtration rate; ELOS, extended length of hospital stay; NLR, neutrophil-to-lymphocyte ratio; NTAR, NT-proBNP-to-albumin ratio; PAH, pulmonary arterial hypertension; RDW, red cell distribution width; RGR, red cell distribution width-to-eGFR ratio.

**Table 5:** Predictors for in-hospital mortality in PAH and CTEPH groups.

Independent Variables	In-Hospital Mortality					
	AUC (95% CI)	*p*	OR (95% CI)	*p*	Hosmer–Lemeshow Test, *p*	Associated Criterion to Youden Index J
**PAH group**						
Prior studied biomarkers						
Albumin	0.804 (0.731–0.865)	<0.001	0.152 (0.037–0.619)	0.008	0.490	≤4.16
NLR	0.858 (0.791–0.910)	<0.001	1.492 (1.163–1.913)	0.001	0.480	>3.28
Log NT-proBNP	0.784 (0.709–0.848)	<0.001	10.291 (1.490–71.065)	0.018	0.498	>3.65
RDW	0.700 (0.619–0.773)	0.054	1.050 (0.993–1.111)	0.082	0.018	>58.60
eGFR	0.701 (0.621–0.774)	0.031	0.974 (0.948–1.000)	0.058	0.411	≤97.39
Proposed biomarkers						
RGR	0.765 (0.688–0.830)	<0.001	1.018 (0.799–1.298)	0.881	0.458	>0.65
NTAR	0.815 (0.743–0.874)	<0.001	12.083 (1.743–83.748)	0.011	0.093	>3.08
**CTEPH group**						
Prior studied biomarkers						
Albumin	0.772 (0.690–0.842)	0.010	0.455 (0.179–1.156)	0.098	0.305	≤3.77
NLR	0.706 (0.619–0.784)	0.079	1.197 (0.869–1.649)	0.268	0.372	>2.96
Log NT-proBNP	0.799 (0.718–0.865)	0.005	13.824 (0.620–308.177)	0.097	0.738	>3.40
RDW	0.578 (0.487–0.665)	0.569	1.016 (0.903–1.144)	0.780	0.214	>55.1
eGFR	0.644 (0.555–0.727)	0.492	0.988 (0.952–1.026)	0.546	0.470	≤49.46
Proposed biomarkers						
RGR	0.637 (0.547–0.721)	0.511	4.229 (1.172–15.264)	0.027	0.434	>1.11
NTAR	0.817 (0.739–0.880)	0.005	7.779 (0.959–63.066)	0.054	0.577	>3.24

AUC, area under the curve; CI, confidence interval; CTEPH, chronic thromboembolic pulmonary hypertension; eGFR, estimated glomerular filtration rate; NLR, neutrophil-to-lymphocyte ratio; NTAR, NT-proBNP-to-albumin ratio; PAH, pulmonary arterial hypertension; RDW, red cell distribution width; RGR, red cell distribution width-to-eGFR ratio.

**Table 6:** Predictors for 3-month all-cause mortality in PAH and CTEPH groups.

Independent Variables	3-Month All-Cause Mortality					
	AUC (95% CI)	*p*	OR (95% CI)	*p*	Hosmer–Lemeshow Test, *p*	Associated Criterion to Youden Index J
**PAH group**						
Prior studied biomarkers						
Albumin	0.782 (0.707–0.846)	0.006	0.135 (0.030–0.604)	0.008	0.686	≤3.88
NLR	0.608 (0.525–0.688)	0.342	1.060 (0.784–1.435)	0.701	0.068	>3.47
Log NT-proBNP	0.564 (0.480–0.646)	0.601	1.340 (0.453–3.958)	0.596	0.324	>3.70
RDW	0.547 (0.463–0.629)	0.680	0.983 (0.901–1.072)	0.703	0.428	≤47.10
eGFR	0.599 (0.515–0.678)	0.399	1.009 (0.988–1.030)	0.382	0.923	>74.63
Proposed biomarkers						
RGR	0.598 (0.514–0.677)	0.396	1.578 (0.009–5.800)	0.374	0.387	≤0.63
NTAR	0.578 (0.494–0.658)	0.508	1.506 (0.501–4.527)	0.465	0.897	>2.36
**CTEPH group**						
Prior studied biomarkers						
Albumin	0.833 (0.757–0.894)	<0.001	0.487 (0.169–1.405)	0.183	0.799	≤3.98
NLR	0.875 (0.805–0.927)	<0.001	1.491 (1.047–2.123)	0.026	0.925	>4.83
Log NT-proBNP	0.743 (0.658–0.817)	0.253	4.929 (0.360–67.448)	0.232	0.117	>4.16
RDW	0.581 (0.490–0.668)	0.654	1.033 (0.905–1.180)	0.625	0.512	>46.8
eGFR	0.793 (0.712–0.860)	0.018	0.945 (0.879–1.017)	0.136	0.822	≤59.21
Proposed biomarkers						
RGR	0.741 (0.655–0.814)	0.081	3.347 (0.807–13.882)	0.095	0.578	>0.78
NTAR	0.748 (0.670–0.816)	0.142	4.232 (0.525–34.083)	0.175	0.277	>3.52

AUC, area under the curve; CI, confidence interval; CTEPH, chronic thromboembolic pulmonary hypertension; eGFR, estimated glomerular filtration rate; NLR, neutrophil-to-lymphocyte ratio; NTAR, NT-proBNP-to-albumin ratio; PAH, pulmonary arterial hypertension; RDW, red cell distribution width; RGR, red cell distribution width-to-eGFR ratio.

## Data Availability

The data presented in this study can be obtained from the corresponding author according to the local and national regulations.

## Abbreviations

The following abbreviations are used in this manuscript:

AUC	Area under the curve
BMI	Body mass index
CHD-PAH	Pulmonary arterial hypertension associated with congenital heart disease
CI	Confidence interval
CKD	Chronic kidney disease
CKD-EPI	Chronic Kidney Disease Epidemiology Collaboration
COPD	Chronic obstructive pulmonary disease
CTD-PAH	Pulmonary arterial hypertension associated with connective tissue disease
CTEPH	Chronic thromboembolic pulmonary hypertension
DVT	Deep vein thrombosis
eGFR	Estimated glomerular filtration rate
ELOS	Extended length of hospital stay
ESC/ERS	European Society of Cardiology/European Respiratory Society
GDPR	General Data Protection Regulation
IQR	Interquartile range
IPAH	Idiopathic pulmonary arterial hypertension
LOS	Length of hospital stay
NLR	Neutrophil-to-lymphocyte ratio
NT-proBNP	N-terminal pro-brain natriuretic peptide
NTAR	NT-proBNP-to-albumin ratio
OR	Odds ratio
PAH	Pulmonary arterial hypertension
PH	Pulmonary hypertension
PoPH	Pulmonary arterial hypertension associated with portal hypertension
RDW	Red cell distribution width
REVEAL	Registry to Evaluate Early and Long-term Pulmonary Arterial Hypertension Disease Management
RGR	Red cell distribution width-to-estimated glomerular filtration rate ratio
ROC	Receiver operating characteristic
SD	Standard deviation
T2DM	Type 2 diabetes mellitus
WHO-FC	World Health Organization functional classification
WBC	White blood cell
